# Effect of Fetal Hypothyroidism on Cardiac Myosin Heavy Chain Expression in Male Rats

**DOI:** 10.5935/abc.20160099

**Published:** 2016-08

**Authors:** Nasibeh Yousefzadeh, Sajad Jeddi, Mohammad Reza Alipour

**Affiliations:** 1 Drug Applied Research Center, Tabriz University of Medical Sciences, Tabriz, Iran; 2 Endocrine Physiology Research Center, Research Institute for Endocrine Sciences, Shahid Beheshti University of Medical Sciences, Tehran - Iran

**Keywords:** Hypothyroidism, Fetus, Cardiac Myosins, Myocardial Contraction, Thyroid Hormones, Rats

## Abstract

**Background:**

Thyroid hormone deficiency during fetal life could affect the cardiac function in later life. The mechanism underlying this action in fetal hypothyroidism (FH) in rats has not been elucidated thus far.

**Objective:**

The aim of this study is to evaluation the effect of FH on cardiac function in male rats and to determine the contribution of α-myosin heavy chain (MHC) and β-MHC isoforms.

**Methods:**

Six pregnant female rats were randomly divided into two groups: The hypothyroid group received water containing 6-propyl-2-thiouracil during gestation and the controls consumed tap water. The offspring of the rats were tested in adulthood. Hearts from the FH and control rats were isolated and perfused with langendroff setup for measuring hemodynamic parameters; also, the heart mRNA expressions of α- MHC and β-MHC were measured by qPCR.

**Results:**

Baseline LVDP (74.0 ± 3.1 vs. 92.5 ± 3.2 mmHg, p < 0.05) and heart rate (217 ± 11 vs. 273 ± 6 beat/min, p < 0.05) were lower in the FH rats than controls. Also, these results showed the same significance in ±dp/dt. In the FH rats, β-MHC expression was higher (201%) and α- MHC expression was lower (47%) than control.

**Conclusion:**

Thyroid hormone deficiency during fetal life could attenuate normal cardiac functions in adult rats, an effect at least in part due to the increased expression of β-MHC to α- MHC ratio in the heart.

## Introduction

Previous studies have shown that several of the diseases taking place in adulthood such as diabetes, hyperlipidaemia, and cardiovascular diseases are the result of intrauterine growth restriction (IUGR).^1^ IUGR can result from a number of causes including malnutrition, stress, and endocrine disorder such as thyroid abnormality.^2,3^

Thyroid hormones have an important role in the development and growth of various organs during life, especially in fetal and neonatal periods.^4^ It has been reported that decrease of thyroid hormones levels during fetal life (fetal hypothyroidism) in rats causes IUGR and induces cardiac dysfunction in the later life of the offspring;^5,6^ however, the underlying mechanisms have not been elucidated yet.

Myosin heavy chain (MHC) is the major contractile protein of the heart tissue and primary regulator of cardiac function and contractility, which represents two MHC isoforms: α-MHC and β-MHC.^7,8^ The proportion of α-MHC and β-MHC isoforms may differ according to development stage, and changes in this ratio hinder cardiac contractility^7^. Increased expression of heart β-MHC, a common feature of heart failure, can be induced by mechanical stress and hypothyroidism.^9,10^

To the best of our knowledge, there is no documented report addressing the mechanism of heart failure in fetal hypothyroidism (FH) rats; the aim of this study is therefore to determine whether changes in the expressions of α-MHC and β-MHC genes are involved in cardiac dysfunction in fetal hypothyroidism rats.

## Methods

### Animals

Male and female Wistar rats were housed in an animal room with a temperature of 22 ± 3°C, relative humidity of 50 ± 6%, and had free access to standard rat chow and tap water during the study. The animals were adapted to an inverse 12:12 h light/dark cycle. All experimental procedures employed, as well as rat care and handling, were in accordance with guidelines provided by the local ethics committee.

### Fetal hypothyroidism induction

Six virgin female Wistar rats (body weight = 200 ± 10 g) were housed overnight with male rats (body weight = 300 ± 20 g) (two female and one male rat per cage) in the proestrus phase determined by vaginal smears for mating. The pregnant females were randomly divided into: 1 - The 0.025% 6-propyl-2-thiouracil (PTU)-consuming during pregnancy and 2-The control mother groups. After this division, they were then transferred to separate cages. The PTU-consuming mothers received PTU in drinking water during pregnancy (initiated on day 1 of pregnancy and discontinued after delivery) and the control mothers received only tap water. After weaning, the male offspring of the mothers were separated and divided into 2 as follows: FH (n = 8) and controls (n = 8), and were housed in groups of four per cage with free access to food and water. In this study, the rats in the control and FH groups were divided into two subgroups and, then, the functional study and molecular analysis were performed. After the birth, the weight of the offspring in all the groups was measured monthly from the first day of birth until the end of the month 3. In addition, weight gain until 3 months of age was measured in the control and FH groups.

### Total T3 and T4 measurements

To assess the thyroid function status, the blood samples were obtained - from the mothers after delivery and the from offspring at birth and 3 months old - centrifuged (3000 × g, 10 min at 4°C), and the sera were stored at -80°C until the time of assay. Total triiodothyronine (TT3) and total thyroxine (TT4) levels were measured by ELISA kits (Pishtazteb Company, Iran). Intra- and inter-assay coefficients of variations were 3.6 and 4.7% for T3 and 5.8 and 6.3% for T4, respectively.

### Measurement of hemodynamic parameters

All the rats at 3 months of age were anesthetized by the intra-peritoneal injection of ketamine and xylazine (60 mg/kg and 10 mg/kg). The hearts of the control and FH rats were rapidly excised and immersed in an ice-cold perfusion buffer; aortas were then cannulated and the hearts were fixed on the constant-pressure mode of the Langendorff perfusion apparatus and perfused through the aorta with a Krebs-Henseleit solution (pH = 7.4) containing (mmol/L): NaCl 118, NaHCO_3_ 25, KCl 4.7, MgCl_2_ 1.2, CaCl_2_ 2.5, KH_2_PO_4_ 1.2, and glucose 11; perfusion pressure of the solution was adjusted constantly at 75 mmHg and Krebs solution was gassed with the mixture of 95% O_2_ and 5% CO_2_ at 37°C.

A latex balloon was inserted in the left ventricle to allow for the measurement of the left ventricular developed pressure (LVDP), peak rates of positive (+dP/dt) and negative (-dp/dt), changes in the left ventricular pressure, and heart rate (HR). Hemodynamic parameters (HR, LVDP, and ±dp/dt) were digitalized by a Power Lab (AD instrument, Australia) system.^11^

### RNA isolation, cDNA synthesis and Real-time quantitative PCR

Total RNA was extracted from the left ventricle of hearts using RNX-Plus solution kit (Fermentase, Cinagen Co. Iran) in accordance to the manufacturer's instructions. RNA quantity and purity were measured using the NanoDrop 1000 (Thermo Scientific, Waltham, and Mass). The α-MHC and β-MHC genes expression were quan titatively assessed by real-time polymerase chain reaction, primers' sequences for each gene were chosen using Gene-Runner Software, version 3.05 (Table 1). For synthesis of cDNA, total RNA was reverse transcribed by means of Revert Aid M-MuLV reverse transcriptase, dNTPS, random hexamer primers, DNase I, and RiboLock RNase-inhibitor, for 10 min at 25°C, followed by 60 min at 42°C in a final volume of 20 µl. The reaction was terminated by heating at 70°C for 5 min.

**Table 1:** The primers sequences

Genes	Primers Sequence^a^
α-MHC	F: GCTGGAGCTGATGCACCTGT
	R: TCGGCATCTGCCAGGTTGTC
β-MHC	F: TCGGGAAGCAGTGCCAGAAC
	R:AGGAGCAGGAAGGGTCGGTT
β-actin	F: TACAGCTTCACCACCACAGC
	R: ATGCCACAGGATTCCATACC

Sequences were derived from NCBI (www.ncbi.nlm.nih.gov).

A master mix containing 12.5 µl SYBR Green PCR Master Mix (Fermentase, Germany), 8.5 µl water and 2 µl forward primer and reverse primer in a final volume of 25 µl was prepared to carry out real-time PCR. Two microliters of reverse transcribed cDNA were then added to the PCR master mix to achieve a final volume of 25 µl. Reactions with no template were included as negative controls.

The PCR protocol was used on the real-time PCR machine (Rotor-Gene 3000) in three steps including: 1 - initial denaturation (10 min at 95°C); 2 - a three-step amplification program (15 s at 95°C followed by 30 s at 60°C for α-MHC and β-MHC genes; and 30s at 72°C) repeated 40 times; and step 3-melting curve analysis (1 cycle: 72 to 95°C with temperature transition rate 1°C/sec for 5 sec). Real-time quantification was monitored by measuring the increase in fluorescence caused by binding of the SYBR Green dye to double-stranded DNA at the end of each amplification cycle. All runs were performed in duplicates. The relative amount of mRNA for each target gene was calculated based on its threshold cycle (Ct) compared to the Ct of the house keeping (reference) gene (β-actin). The relative quantification was performed by 2^-∆ ∆ Ct^ method as follows:^12^

2-[(Ct target gene - Ct reference gene) experimental - (Ct target gene -Ct reference gene) Control]. The specificity of the PCR reactions was verified by generation of a melting curve analysis.

### Statistical analysis

All the values were expressed as means ± SEM. The statistical analysis was performed using SPSS software (SPSS, Chicago, IL, USA; version 20). Shapiro-Wilk test was used to check the normality of the studied data and, then, parametric or non-parametric tests were used for the analysis of normal or non-normal data distribution, respectively.^13^ Therefore, the *Student's sample t-test* was used to compare thyroid hormone levels, LVDP, HR, ± dp/dt, and body weight between the groups. Mann-Whitney U test was also used for comparing gene expression between the two groups. Two-sided p values of < 0.05 were considered statistically significant.

## Results

**Figure 1:**
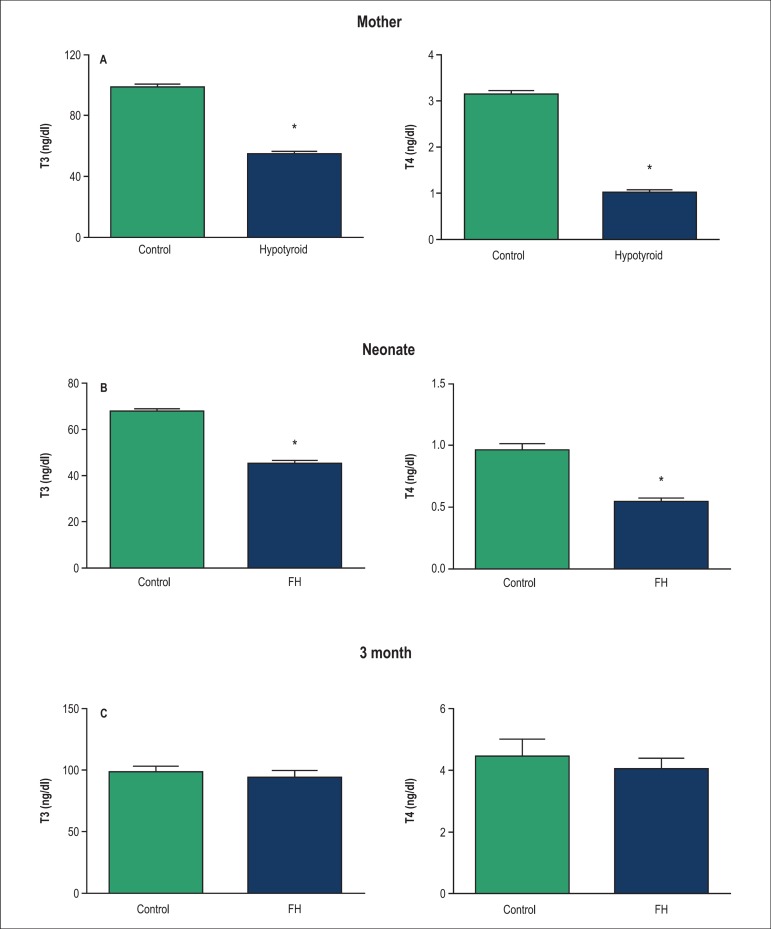
Serum T 3 and T 4 concentrations in mother (A), neonate (B) and 3 month of age (C) in the fetal hypothyroid, and control rats. Values are mean ± SEM. *p < 0.05, statistically significant differences between hypothyroid and control in mothers and fetal hypothyroid and control rats in offspring. (n = 8 rats). T_4_: Thyroxine; T_3_: triiodothyronine; FH: Fetal hypothyroidism.

Effects of PTU administration on thyroid hormone levels during pregnancy (total T4 and T3), in serum from the mothers are shown in Figure 1A. As shown, the mothers which consumed PTU during pregnancy had lower total T4 and T3 in serum compared with the control group (p < 0.05).

Total T4 and T3 in serum from the offspring after the administration of PTU in pregnant mothers are shown in Figure 1B and 1C. The offspring at birth had lower total T4 and T3 in serum compared with the control group (p < 0.05) (Figure 1 B), whereas in adulthood (month 3), there was no significant difference between the two groups (Figure 1C).

**Figure 2:**
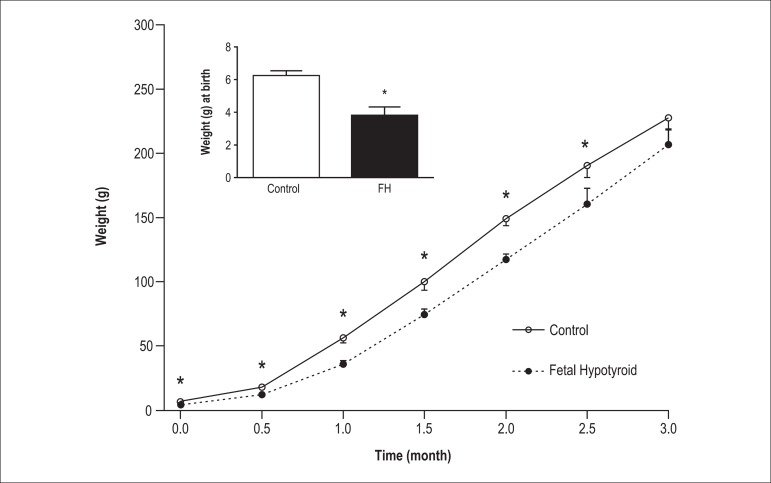
Body weight in the fetal hypothyroid and control rats. Values are mean ± SEM. *p < 0.05, statistically significant differences between fetal hypothyroid and control rats. (n = 8 rat). FH: Fetal hypothyroidism.

Figure 2 shows the effects of thyroid hormone deficiency during fetal life on body weight in the offspring (at birth until 3 months of age). As shown, body weight at birth was significantly lower in the FH rats than the controls. In addition, weight gain until 3 months of age was significantly less in the FH rats compared with the controls.

**Figure 3:**
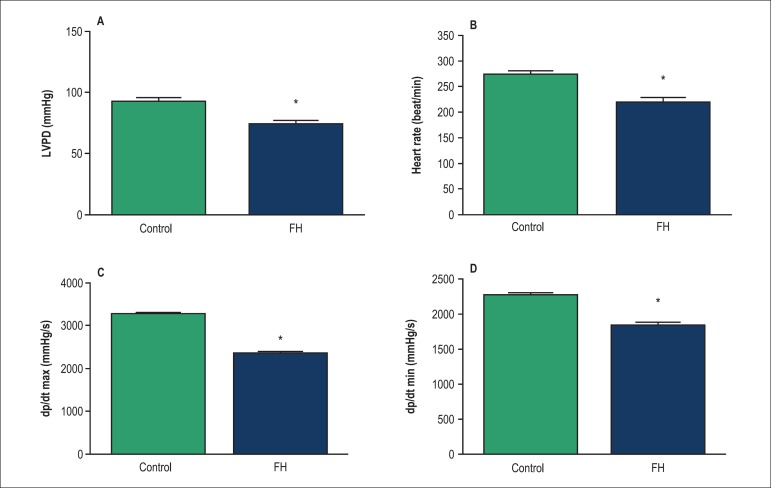
Hemodynamic parameters in the fetal hypothyroid and control rats. A) Left ventricular developed pressure (LVDP); B) Heart rate; C) Peak rates of positive changes in left ventricular pressure (+dp/dt); D) Peak rates of negative changes in left ventricular pressure (-dp/dt); Values are mean ± SEM; (n = 8 rats); *p < 0.05, statistically significant differences between fetal hypothyroid and control rats. FH: Fetal hypothyroidism.

Hemodynamic parameters including LVDP, ±dp/dt and heart rate in the offspring (at 3 months of age) which had thyroid hormone deficiency during fetal life are demonstrated in Figure 3. The baseline level of LVDP in the FH rats was significantly lower than that of the controls (Figure 3A) (p < 0.05). In addition, the FH rats had lower baseline levels of the heart rate and +dp/dt and -dp/dt than the controls (Figures 3B, 3C and 3D, respectively) (p < 0.05).

**Figure 4:**
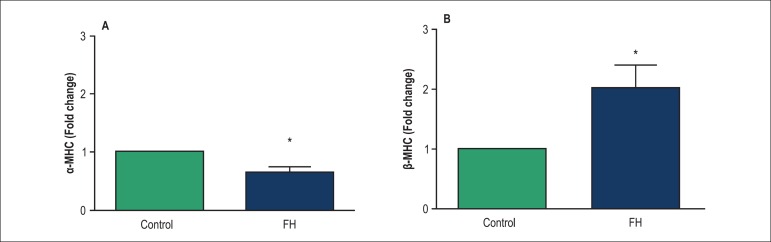
Gene expression in hearts of the fetal hypothyroid and control rats. MHC; (myosin heavy chain) *p < 0.05. Data were normalized to the calibrator (set to 1) and presented as mean ± SEM. FH: Fetal hypothyroidism.

Effects of thyroid hormone deficiency during fetal life on the expression of β- and α-MHC expressions in the offspring with 3 months of age are shown in Figure 4. In the FH rats, β-MHC expression was higher (201%) (Figure 4A) (p < 0.05) and α- MHC expression was lower (47%) compared with control rats (Figure 4B) (p < 0.05).

## Discussion

Our results showed that thyroid hormones deficiency in fetal life could attenuate the normal cardiac functions including LVDP, HR, and ±dp/dt during adulthood. Cardiac dysfunction in FH rats is, at least in part, due to increased β-MHC expression and decreased α-MHC expression.

In this study, the FH rats had less weight gain until month 3 of age, a result in line with previous studies.^6,13^ Previous reports have shown that low birth weight is a risk factor for later diseases in adulthood in humans and animals, including cardiovascular diseases.^2,14-15^

Previous reports have also demonstrated that the cardiovascular functions can be affected by IUGR.^5^ In the present study, PTU consumption during the pregnancy period in mothers induced IUGR in the offspring. It has been reported that thyroid hormones deficiency in fetal life causes IUGR.^6^ Results from this study demonstrated that hypothyroid mothers at the time of delivery and their offspring at birth had lower thyroid hormones levels (total T3 and T4 levels) compared with the corresponding controls. However, the levels of these hormones in the adult offspring were normal, indicating that the FH rats had thyroid hormones deficiency only during the fetal period. Previous studies have indicated that thyroid hormones are necessary for the development and normal cardiac function during life, especially fetal life.^6,16^

In this study, the FH rats had lower baseline level of LVDP, ±dp/dt, and heart rate compared to controls. Decreases observed in LVDP, ±dp/dt, and also in the heart rate in this study were similar to those of the previous reports,^6^ findings which show that thyroid hormones deficiency in fetal life could influence normal cardiac function and induce cardiac dysfunction in their adult offspring. In addition, pervious reports have demonstrated that lower baseline hemodynamic parameters and cardiac failure are the common outcomes of adult hypothyroidism.^11,17^

The mechanisms of the effects of thyroid hormones deficiency in fetal life on cardiac development and function during adulthood have not been clearly elucidated. In this study, we showed for the first time that thyroid hormones deficiency in fetal life is associated with increased β-MHC expression [2.11 fold higher expression (201%) than control] and decreased α-MHC expression [0.63 fold lower expression (47%) than control]. No report has however been documented regarding the changes in α- and β-MHC expression in FH rats; previous reports have represented that the induction of hypothyroidism during adult life leads to a shift from α- to β-MHC.^9,10,18^ Increased expression of β-MHC in the heart tissue, a frequent aspect of cardiac failure, may decrease power output and lead to decreased systolic function in the heart.^19^ α-MHC had a fast ATPase activity and higher velocity of fiber shortening than β-MHC, so α-MHC: β-MHC ratio in the heart can determine cardiac contractility.^20^ Hypothyroidism in adult rats leads to the increase expression of β-MHC and decrease velocity of fiber shortening and, then, diminishes cardiac contractility.^20,21^ Chizzonite R.A. et al. reported that an increase in α-MHC observed after birth was concurrent with a remarkable increase in the serum level of thyroid hormones.^18^

Of other possible molecular mechanisms involved in the effect of FH on cardiac function, Chizzonite et al.^18^ reported that fetal hypothyroidism delayed the shift of β-MHC (embroyonic MHC in rats) to α-MHC (adult MHC in rats) and caused cardiac disability. Wibo et al.^22^ also reported that the maturation of dihydropyridine receptor (a voltage L type calcium channel) was postponed after the induction of hypothyroidism in fetal and neonate rats and induced ionic imbalance in the heart tissue. In addition, Meehan et al.^23^ reported that thyroid hormone deficiency in fetal life decreased the level of energy in cardiac cells due to the reduction in the expression of cytochrome c oxidase isoforms and vital enzyme for the production of energy in the electron transport chain in the heart tissue during development period. According to the result from this study and previous reports, it is hence possible for the decrease of cardiac function in the FH rats to be related to the increased expression of β- to α- MHC ratio and deregulation in cytochrome c oxidase isoforms expression and dihydropyridine receptor, all of which could contribute to the power of cardiac function.

Regarding the limitations of this study, our results were limited to male rats, and fetal hypothyroidisms could affect cardiac functions in both sexes. In addition, we used the Langendorff-perfused heart model for the measurement of cardiac function; it has been however reported that using in vivo model is clinically more relevant.

## Conclusion

Thyroid hormone deficiency during fetal life could attenuate the normal cardiac functions in adult rats, an effect at least in part due to the increased expression of β- to α- MHC ratio in the heart.
